# Supplementary material for: Targeting Stage-Specific Embryonic Antigen 4 (SSEA-4) in Triple Negative Breast Cancer by CAR T Cells Results in Unexpected on Target/off Tumor Toxicities in Mice
Source: Int J Mol Sci. 2023 May 24;24(11):9184. doi: 10.3390/ijms24119184 (PMC10252441; doi:10.3390/ijms24119184)
Supplement: Supplementary file 1 [file ijms-24-09184-s001.zip › ijms-2372282-supplementary.pdf]

Supplements

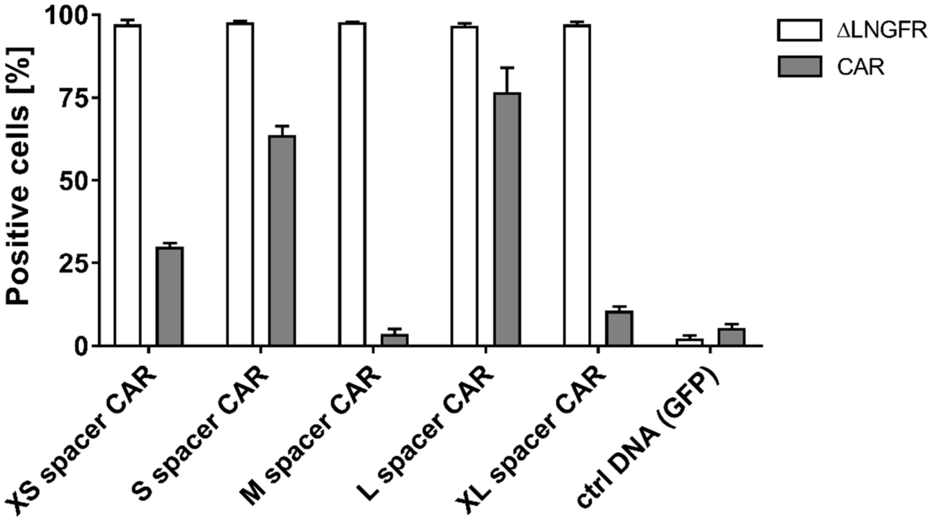

Figure S1: Expression of ΔLNGFR and second generation XS, S, M, L, and XL spacer CAR variants on HEK293T cell surface. Transfer vector DNA was introduced transiently into HEK293T cells by lipofection and flow cytometric analysis was performed 48 hours later. Expression of ΔLNGFR was detected by mAb staining, while that of CARs by Protein L staining. Both detection reagents were used in biotinylated form and fluorescence tagging was performed by secondary α-Biotin-APC mAb labeling. A GFP-encoding vector served as control. Results are the average of 3 independent experiments.

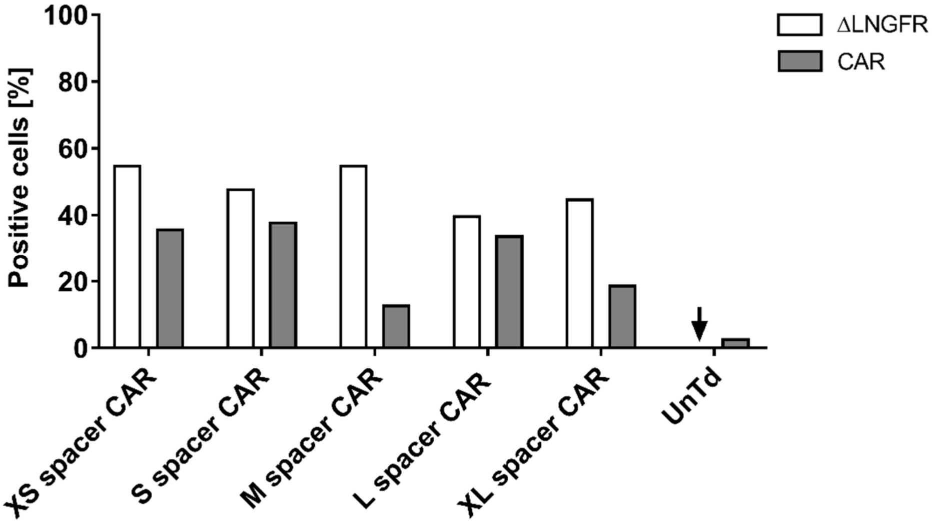

Figure S2: Expression of ΔLNGFR and second generation XS, S, M, L, and XL spacer CAR variants on T cell surface. Naïve Pan T cells were isolated from PBMCs and cultured in the presence of IL-2 and the T cell stimulating matrix TransAct. For permanent receptor expression, the lymphocytes were transduced lentivirally with an MOI of 1.5 and expanded in IL-2 (40 IU/mL). Surface transgene expression was determined by flow cytometry 5 days after transduction and 4 days after TransAct removal, respectively. Expression of ΔLNGFR was assessed y mAb-based staining, while Protein L staining was applied to analyze CAR expression. Both detection reagents were used as biotin-conjugates and fluorescence marking was achieved by secondary labeling with α-Biotin-APC mAb. The results of one experiment are displayed. UnTd, untransduced.

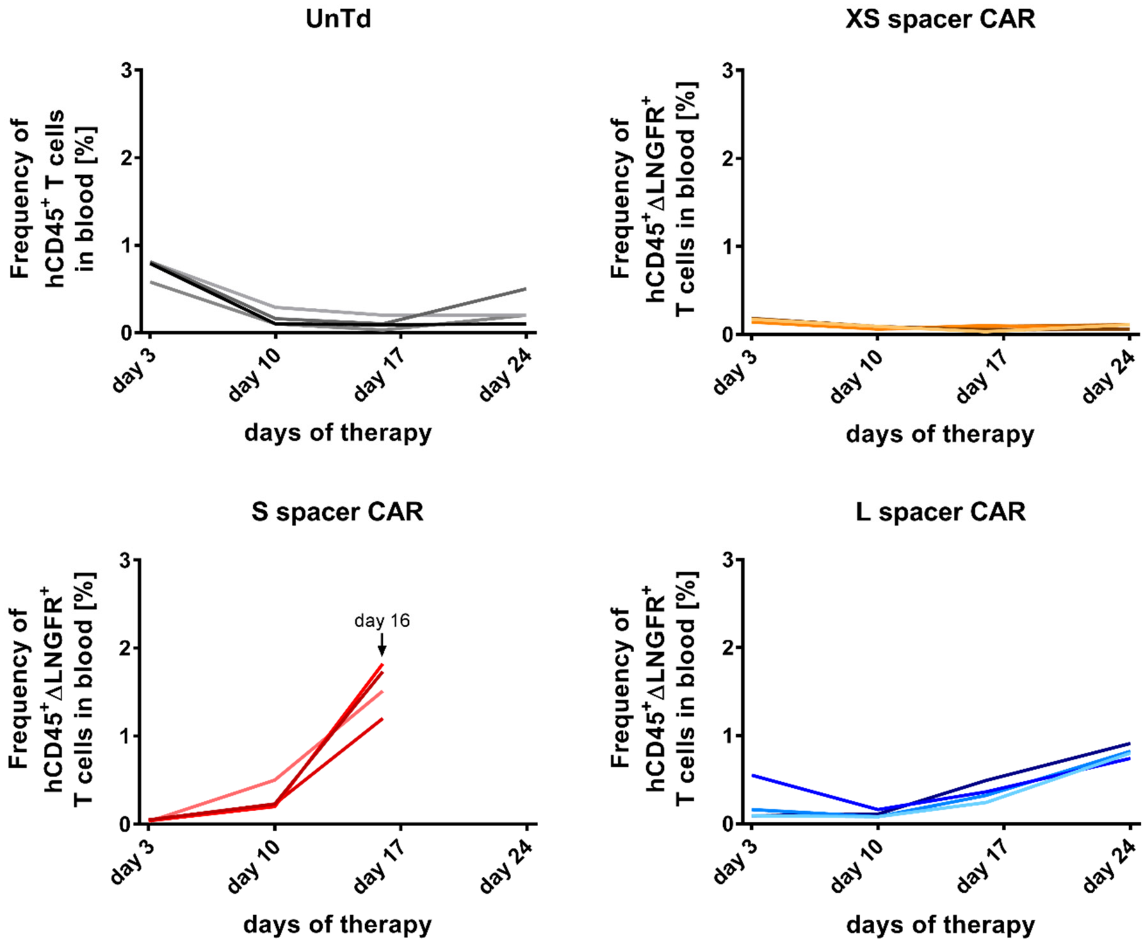

Figure S3: Frequency of CAR (human CD45$^+$ΔLNGFR$^+$) and untransduced (human CD45$^+$) control T cells in mouse peripheral blood over the course of therapy. Female NSG mice with established s.c. MDA-MB-231 were injected i.v. with 2×10^6 XS, S, and L spacer CAR T cells and starting day 3 post adoptive transfer their frequency in peripheral blood was monitored by flow cytometry. For injection of untransduced control T cells, cell number was adjusted to the highest total T cell number injected in the CAR T cell groups. Each line represents one mouse.

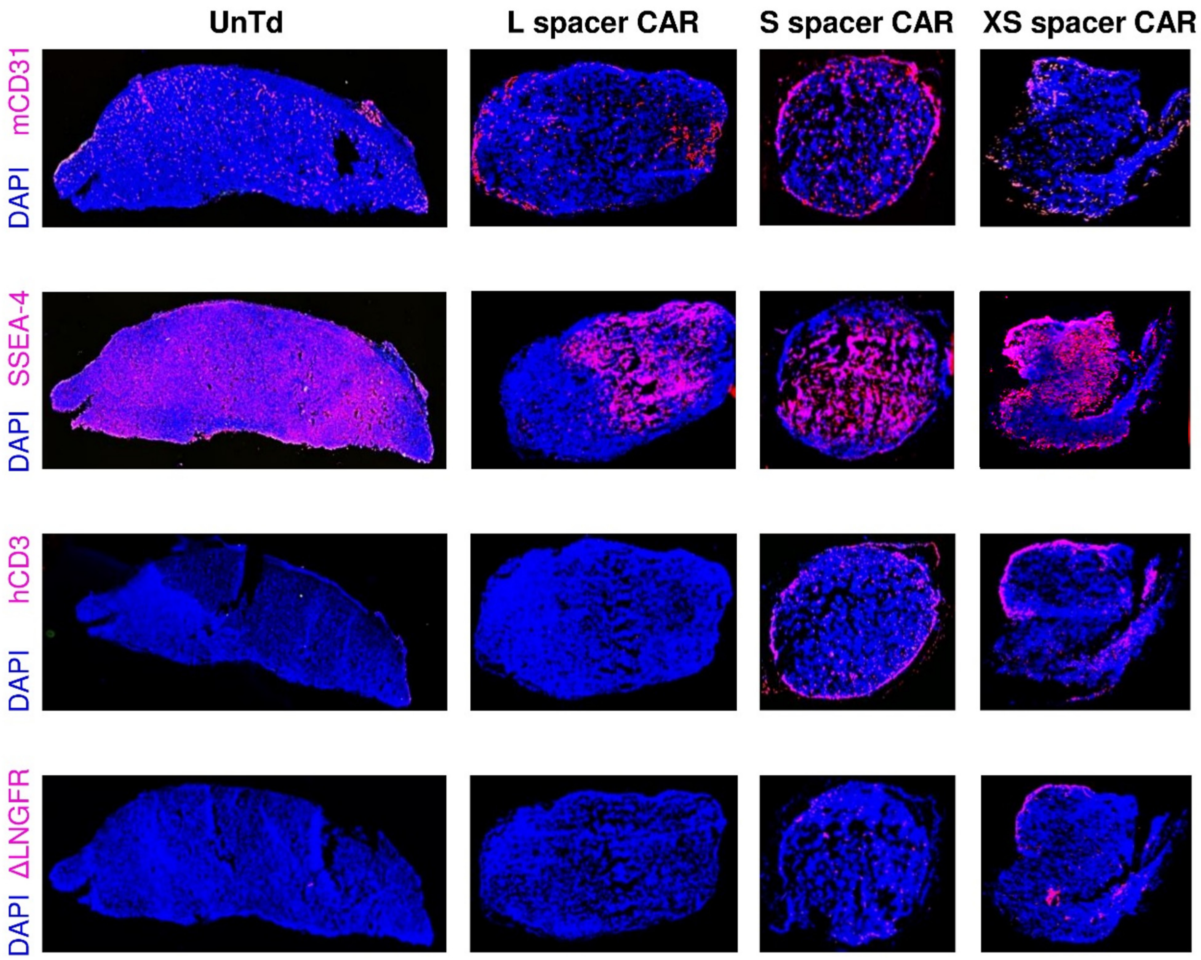

Figure S4: Characterization of MBA-MB-231 xenograft tumors from NSG mice after treatment with L, S, XS spacer CAR or untransduced controls T cells. All images were acquired from tumors treated with $2*10^6$ CAR T cells using a 4x objective lens and are representative for 2 tumors analyzed per group. Pink: mouse CD31, SSEA-4, human CD3, or ΔLNGFR; blue: DAPI.
